# Amino Acid Residues 68–71 Contribute to Influenza A Virus PB1-F2 Protein Stability and Functions

**DOI:** 10.3389/fmicb.2017.00692

**Published:** 2017-04-21

**Authors:** Yi-Ying Cheng, Shih-Rang Yang, Ying-Ting Wang, Yu-Hsin Lin, Chi-Ju Chen

**Affiliations:** ^1^Institute of Microbiology and Immunology, National Yang-Ming UniversityTaipei, Taiwan; ^2^Program in Molecular Medicine, National Yang-Ming University and Academia SinicaTaipei, Taiwan

**Keywords:** influenza A virus, PB1-F2, protein stability, mitochondrial localization, interferon antagonism

## Abstract

Influenza A virus PB1-F2, encoding a multi-functional protein, is regarded as a virulent gene. Variation in expression pattern and protein stability among PB1-F2 proteins derived from different strains may explain why PB1-F2 functions in a strain- and cell type-specific manner. Because the protein stability of PB1-F2 affects its biological functions, we looked for sequences important for this property. By comparing variants and chimeric of PB1-F2 proteins from A/Hong Kong/156/1997 (H5N1) and A/Puerto Rico/8/1934 (H1N1), we identified amino acid residues 68–71 affect its protein stability. PB1-F2 with T68, Q69, D70, and S71 has a shorter protein half-life than its I68, L69, V70, and F71 counterpart. This is likely to do with proteasome-mediated degradation. Swapping amino acids 68–71 between two proteins reversed not only the length of protein half-life and sensitivity to MG132, but also subcellular localization and interferon antagonization. Our data suggested that composition of amino acids 68–71, which regulates protein stability and therefore its functions, can be a major factor determining strain-specificity of PB1-F2.

## Introduction

Influenza A virus (IAV), belonging to the family *Orthomyxoviridae*, is a highly contagious respiratory pathogen that causes diseases in human and animals worldwide. Epithelial cells lining the respiratory mucosa are the main targets of IAV, while macrophages and dendritic cells residing the respiratory tract can also be infected. IAV-associated morbidity and mortality are often caused by severe inflammation characterized by cytokine storm and acute respiratory distress syndrome. Consistently it is found that highly pathogenic IAV strains caused sever lung damage is due to immunopathogenesis in animal models. Its eight-segmented single-stranded RNA genome can encode up to 17 proteins though RNA splicing or leaky scanning (Palese, 1977; Chen et al., 2001; Wise et al., 2009, 2012; Jagger et al., 2012; Muramoto et al., 2013). Among them, PB1-F2 is a small protein encoded by the +1 open reading frame (ORF) in the PB1 gene (Chen et al., 2001).

PB1-F2 was originally identified in a searching for CD8+ T cell epitopes encoded by IAV and its pro-apoptotic activity in A/Puerto Rico/8/1934 (H1N1) (herein PR8)-infected U937 cells (Chen et al., 2001). It induces apoptosis in host immune cells (Chen et al., 2001; Lowy, 2003) via interacting with two mitochondrial proteins, ANT3 and VDAC1, resulting the loss of mitochondria membrane potential (Varga et al., 2011, 2012). In addition, aggregated PB1-F2 from pathogenic strain IAV can be incorporated into the phagolysosomal compartment to activate the NLRP3 inflammasome, resulting IL-1β secretion (Ichinohe et al., 2010; McAuley et al., 2013). Expression of PB1-F2 promotes secondary bacterial infection, increased macrophage and neutrophil lung infiltration, and cytokine deregulation in mice (McAuley et al., 2007). Therefore, it is regarded as a virulent gene. On the other hand, binding of PB1-F2 of PR8 to MAVS, an RIG-I-like receptor (RLR)-signaling adaptor anchoring to mitochondria, leads to antagonism activity on interferon production (Varga et al., 2011, 2012). How the multi-functions possessed by PB1-F2 play in modulating IAV induced immunopathogenesis remains elucidative.

The functions of PB1-F2 are strain-specific and cell type-specific (Varga and Palese, 2011). For instance, PB1-F2 from A/Hong Kong/156/1997 (H5N1) (herein HK156) exhibits cytoplasmic and nuclear localization, a distinctive feature when compared to mitochondrial localization of PB1-F2 of PR8 (Chen et al., 2010). Its cell-type specificity is evident that it induces apoptosis in monocytes but not in epithelial cells (Chen et al., 2001). Not all influenza viruses express full-length PB1-F2 though; it is conserved in avian influenza virus strains, but is lost in many isolates from mammalian hosts (Schmolke et al., 2011). Up to date, most human H1N1 isolates produce truncated PB1-F2 (57 amino acids) or none, while human H3N2 isolates or avian H5N1 isolates produce full-length PB1-F2 (87–90 amino acids) (Chakrabarti and Pasricha, 2013). One study using a 27-mer PB1-F2-derived from C-terminal amino acids 61–87 of PB1-F2 (PR8) caused severe inflammation and predisposed mice to secondary bacterial infection, suggesting the last one third of PB1-F2 protein contributes to IAV pathogenesis. I68, L69, and V70 are found to be essential for this property (Alymova et al., 2014). It is not fully understood the advantage/disadvantage of expressing this gene or why many human isolates lack it without selective disadvantage. It is equally unclear what determines its strain- and function-specificity.

The kinetics of PB1-F2 production varies when cells are infected with different strains of IAV. PB1-F2 protein was not synthesized at the same level when expression plasmids for PB1-F2 variants were transfected into HEK293T (Chen et al., 2010). Among eight PB1-F2 variants examined by Western analysis, only four including PB1-F2 of PR8, HK156, Taiwan/3355 (H1N1), and Netherlands/219 (H7N7) can be detected with PB1-F2 (HK156) at the lowest level, although messenger RNAs were transcribed at a similar level. Three of four variants that were not detected originally became detectable after radiation labeling following immunoprecipitation or linker addition to increase protein flexibility. These results revealed that once made PB1-F2 is degraded rapidly. Among them, however, only expression of PB1-F2 of HK156 was rescued by MG132, a proteasome inhibitor (Chen et al., 2010), suggesting that PB1-F2 may be degraded via multiple mechanisms. Since it has been demonstrated that degradation propensity is one of the key factors affecting functions of PB1-F2 (Kosik et al., 2013, 2015), we looked for the sequence important for its protein level. Here we established that sequence of amino acid 68–71 of PB1-F2 played a crucial role in regulating its protein half-life. PB1-F2 of HK156 had a much shorter half-life compared to that of PR8. Swapping of amino acids 68–71 between two proteins reversed not only the protein half-life and sensitivity to MG132, but also biological properties of their original counterparts, including subcellular localization and interferon antagonizing function.

## Materials and Methods

### Cell Lines and Culture

Human embryonic kidney 293T cells (HEK293T) and HeLa cells were cultured in Dulbecco’s modified Eagle’s medium supplemented with 10% fetal bovine serum, streptomycin, and penicillin at 37°C with 5% CO_2_.

### Plasmids Construction and Mutagenesis

The expression plasmids [backbone: pFLAG-CMV2 (Sigma–Aldrich) for FLAG-tagged PB1-F2 (PR8) and PB-F2 (HK156) were provided by S.-R. Shih (Chang Gung University, Taiwan) (Chen et al., 2010). Plasmid constructions for FLAG-tagged chimeric PB1-F2 (HP1, HP3, and HP3) were done by overlapping PCR extension cloning. For each construct, a pair of flanking primers and either a pair (HP1 and HP3) or two pairs (HP2) of internal primers were used to make the final product containing chimeric PB1-F2 (**Tables 1, 2**). Coding region of each chimeric PB1-F2 was cloned into pFLAG-CMV2 at 5′-*Hin*dIII and 3′-*Eco*RI sites. For making expression plasmids for EGFP-PB1-F2, coding region of each PB1-F2 was amplified by PCR from pFLAG-CMV2-based plasmids, and then inserted into pLNFG (a gift of P.-Y. Wu, Academia Sinica, Taiwan) (Pan et al., 2015) at 5′-*Xho*I and 3′-*Cla*I. Site-directed mutagenesis of amino acids 68–71 was carried out by primer extension. Briefly, segments of PB1-F2 coding region with amino acids 68–71 mutated were amplified from the pLNFG-PB1-F2 (wild type) using flanking primers and internal primers containing desired mutation. PCR products were cloned into pLNFG at 5′-*Xho*I and 3′-*Cla*I sites. Fragments containing coding region of PB1-F2 (PR8-TQDS) and PB1-F2 (HK156-ILVF) were PCR amplified and subcloned from pLNFG-based constructs into pFLAG-CMV2 at 5′-*Hin*dIII and 3′-*Eco*RI sites. Primer design and their uses, as well as the templates are listed in **Tables 1, 2**. All constructs were sequenced for accuracy.

**Table 1:** Primer used for constructing PB1-F2 expressing plasmids.

	Flanking primers		Internal primers		
	Forward	Reverse	Forward	Reverse	Template
pFLAG-HP1	No. 621	No. 622	No. 613	No. 614	pFLAG-PB1-F2 (PR8) pFLAG-PB1-F2 (HK156)
pFLAG-HP2	No. 621	No. 622	No. 615 No. 617	No. 616 No. 618	
pFLAG-HP3	No. 621	No. 622	No. 619	No. 620	
pFLAG-HK156-ILVF	No. 797	No. 798	–	–	pLNFG-HK156-ILVF-F2
pFLAG-PR8-TQDS	No. 795	No. 796	–	–	pLNFG-PR8-TQDS-F2
pEGFP-PB1-F2 (HK156)	No. 675	No. 622	–	–	pFLAG-PB1-F2 (PR8)
pEGFP-PB1-F2 (PR8)	No. 674	No. 622	–	–	pFLAG-PB1-F2 (HK156)
pEGFP-HK156-ILVF	No. 675	No. 622	No. 738	No. 737	pFLAG-PB1-F2 (PR8) pFLAG-PB1-F2 (HK156)
pEGFP-PR8-TQDS	No. 674	No. 622	No. 746	No. 747	
pHW192-PB1-F2-TQES	No. 683	No. 684	No. 766	No. 767	pHW192


*Vector for EGFP-tagged PB1-F2: pLNFG; Vector for FLAG-tagged PB1-F2: pFLAG-CMV2*.

**Table 2:** Primer sequences.

Primer no.	Sequence
No. 613	CCGAAACTGGAGCACCCCAACTCAAC
No. 614	TTGGGGTGCTCCAGTTTCGGTGTTTG
No. 615	CAGAGACCGGAGCACCGCAACTCAAC
No. 616	TTGCGGTGCTCCGGTCTCTGTGTTTG
No. 617	GGAGGCGATGGCTTTCCTTGAAGAATC
No. 618	CAAGGAAAGCCATCGCCTCCAATACAC
No. 619	GGAAGCAATGGCTTTCCTTGAGGAATC
No. 620	CAAGGAAAGCCATTGCTTCCAATACAC
No. 621	CGCAAATGGGCGGTAGGCGTG
No. 622	TGCCCCTTGCTCCATACCACCC
No. 674	*AGATCT*CGAGAG**ATG**GGACAGGAACAGGATACACC (*Xho*I)
No. 675	*AGATCT*CGAGAG**ATG**GAACAGGAACAGGATACACC (*Xho*I)
No. 683	AAC*GCTAGC*AGTTAACCGGAGTAC (*Nhe*I)
No. 684	CTT*GCTAGC*ATTTCTGCAGGTATTTG (*Nhe*I)
No. 737	GTTCTCAAAAATACCAGGATGGGATTCTTCAAGG (ILVF)
No. 738	CCCATCCTGGTATTTTTGAGAACTCATGTC (ILVF)
No. 746	CCACCCAGGACTCTTTGAAAACTCGTGTA (TQDS)
No. 747	GTTTTCAAAGAGTCCTGGGTGGGATTCCTCAAGG (TQDS)
No. 766	CCCACCCAGGAATCTTTGAAAACTCGTG (TQES)
No. 767	GATTCCTGGGTGGGATTCCTCAAGG (TQES)
No. 795	CGAC*AAGCTT***ATG**GGACAGGAACAGG (*Hin*dIII)
No. 796	CGAT*GAATTC***CTA**CTCGTGTTTGCTG (*Eco*RI)
No. 797	CGAC*AAGCTT***ATG**GAACAGGAACAGG (*Hin*dIII)
No. 798	CGATG*AATTC***TTA**GTTTGTCCACTC (*Eco*RI)


*Restriction sites were italicized; mutated sequences were underlined; start codon in forward primers and stop codons in reverse primers are in bold*.

### Transient Transfection

All plasmids for transfection were purified by using the Plasmid Midi kit (QIAGEN). Transfection of HEK293T and HeLa was performed using Lipofectamine 2000 reagent (Invitrogen) or jetPRIME transfection reagent (Polyplus-transfection) according to the manufacturer’s instruction.

### Western Blot Analysis

Total cells were lysed in sample buffer (1% sodium dodecyl sulfate, 1.5% β-mercaptoethanol, 4% glycerol, 0.01% bromophenol blue in 50 mM Tris–HCl), resolved by SDS–polyacrylamide gel, and electro-transferred onto polyvinylidene difluoride membranes. After blocked in Tris–buffered saline (50 mM Tris–HCl [pH 7.4], 0.2 M NaCl and 0.1% Tween 20) containing 5% non-fat milk, the membranes were incubated with various antibodies. Membranes were then washed with TBS-T and incubated with horseradish peroxidase-conjugated secondary antibody. Signals were detected with ECL Western blotting detection reagent (GE Healthcare). Commercial primary antibodies used were anti-FLAG (Sigma), anti-GFP (GeneTex), anti-GAPDH (Novus), anti-β-actin (GeneTex) anti-PB1 (GeneTex), anti-NA (GeneTex), and anti-NS1 (Santa Cruz) antibodies. The house-made rabbit polyclonal anti-PB1-F2 antibody was raised against amino acid residues 2–16 (GQEQDTPWILSTGHI) of PB1-F2 (PR8).

### Cycloheximide Chase Assay and Proteasome Inhibition

**FIGURE 1:**
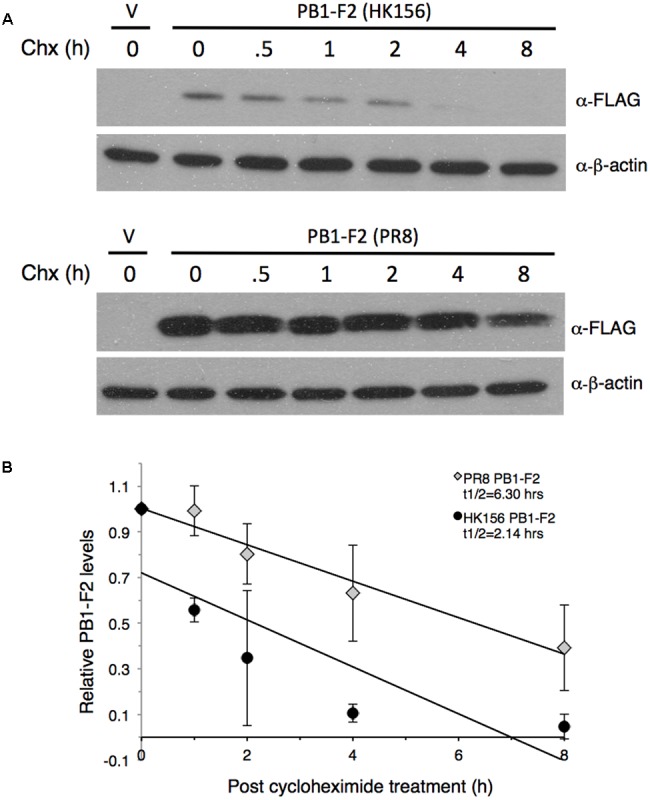
**Measurement of PB1-F2 half-life.** Plasmids expressing FLAG-PB1-F2 (HK156) and FLAG-PB1-F2 (PR8) were transfected into HEK293T cells for 16 h before cycloheximide was added. Cells were harvested at 0, 0.5, 1, 2, 4, and 8 h post-cycloheximide treatment for Western analysis using anti-FLAG antibody. Additional three sets of experiments were performed without 0.5 h time point. **(A)** Representative blots for PB1-F2 (HK156) (Top) and PB1-F2 (PR8) (Bottom) were shown. V, vector control. **(B)** Chemiluminescence intensity for bands specific to PB1-F2 at each time point were captured and measured by a biomolecular imager and plotted after normalized with β-actin. Band intensity at time zero was set as 1. Regression formulation calculated from three sets of experiment is *y* = –0.103*x*+0.7205 for PB1-F2 (HK156) and *y* = –0.0799*x*+1.0031 for PB1-F2 (PR8). Protein half-life (t1/2) for each PB1-F2 was as indicated.

HEK293T cells were transfected plasmids expressing FLAG-tagged PB1-F2 for 16 h before cycloheximide treatment (50 μg/ml). At 0, 1, 2, 4, and 8 h post-cycloheximide treatment, cells were harvested and lysed in SDS sample buffer. An additional 0.5 h time point was added in **Figure 1**. Protein levels of FLAG-PB1-F2 were examined by Western analysis using anti-FLAG antibody. Chemiluminescence intensity for bands specific to PB1-F2 at each time point were captured and measured by a biomolecular imager (ImageQuant LAS 4000). Signals were plotted and analyzed after normalized with β-actin as percentage to time zero using Multi Gauge software (Fujifilm). Band intensity at time zero was set to 100%. The protein half-life (t1/2) for PB1-F2 was calculated from regression formulation taken from three independent experiments. For proteasome inhibition experiments, HEK293T cells were transfected with pFLAG-PB1-F2 or pEGPF-PB1-F2 plasmids. After being transfected for 6–8 h, cells were treated with of MG132 (5 μM) for 16 h to block proteasome function. Chemiluminescence intensity for bands specific to EGFP-PB1-F2 and GAPDH were captured and measured by a biomolecular imager (ImageQuant LAS 4000). Signals with or without MG132 treatment were compared after normalized with GAPDH. Intensity of EGFP-PB1-F2 (MG132 (-)) after normalized with GAPDH was set as 1x.

### Immunofluorescence and Confocal Microscopy

HeLa cells were grown on coverslips before being transfected with individual EGFP-tagged PB1-F2 expression plasmid. At 16 h post-transfection, cells were stained with 250 nM reduced rosamine MitoTracker Orange (M7511, Invitrogen) in HBSS (Gibco), fixed with 4% formaldehyde and then permeabilized with 0.5% Triton X-100. Nuclei were stained with DAPI (Sigma). Confocal microscopy was performed with a Zeiss LSM700 microscope. The images were acquired using Plan-Apochromat 1.4 Oil DIC M27 objectives with power of 63× and 100× with appropriate filter combinations. Ten fields were randomly selected to calculate the percentage of the EGFP-PB1-F2 that colocalized with MitoTracker signals.

### IFN-β Reporter Assay

3 × 10^5^ HEK293T cells were co-transfected with individual EGFP-tagged PB1-F2 expressing plasmid and p125-luc IFN-β luciferase reporter (a gift of L.-H. Hwang, National Yang-Ming University, Taiwan) in 12-well plates. 24 h post-transfection, cells were infected with Sendai virus (SeV) H4 (50 HAU) to induce IFN-β signaling pathway. Cells were harvested 24 h post-infection for luciferase activity using Luciferase Assay System (Promega). Two independent experiments were performed in triplicate for each sample.

### PB1-F2 Production by Recombinant Virus

Recombinant A/Puerto Rico/8/1934 carrying PB1-F2 (68T, 69Q, 70E, 71S) was made using an eight-plasmids reverse genetics system (Hoffmann et al., 2000). Plasmids were kindly provided by Dr. M.-S. Ho (Academia Sinica, Taiwan). Coding region of amino acid residues 68–71 was mutated from ILVF to TQES without affecting the coding of PB1 in plasmid pHW192 using overlapping PCR extension cloning. Primer design and their uses, as well as the templates are listed in **Tables 1, 2**. Resulting plasmid pHW192 or pHW192-(PB1-F2 TQES) was transfected with seven other pHW19x plasmids into HEK293 cells using jetPRIME transfection reagent to generate PR8 wild-type virus or PR8 (PB1-F2-TQES) mutant virus. At 16 h post-transfection, medium was replaced with DMEM without serum. Additional 32 h later, the supernatant was collected and used to inoculate A549 cells. A549 cells were harvested 24 h post-infection for Western analysis using antibodies as indicated.

## Results

PB1-F2 is regarded as a virulent gene with multiple functions. However, its protein product is unstable and can be degraded rapidly through proteasome-dependent machinery (Chen et al., 2010). It has been shown that the expression pattern of PB1-F2 varies among IAV strains (Chen et al., 2010). Since the protein stability of PB1-F2 plays an important role in its functions, we looked for key amino acid residues affecting its protein level.

### Protein Half-life of PB1-F2

To compare the stability of PB1-F2 variants, we measured protein half-lives of PB1-F2 from A/Hong Kong/156/1997 (H5N1) (herein PB1-F2 (HK156)) and A/Puerto Rico/8/1934 (H1N1) (herein PB1-F2 (PR8)) by cycloheximide chase assay. The plasmid expressing either FLAG-PB1-F2 (HK156) or FLAG-PB1-F2 (PR8) was transfected to HEK293 cells for 16 h before cycloheximide was added to stop translation. Cells were harvested at 0, 0.5, 1, 2, 4, and 8 h post-cycloheximide treatment for Western analysis. Intensity for bands specific to PB1-F2 at each time point was measured by densitometer and plotted. A representative Western analysis (**Figure 1A**) and a graph of the mean band intensities and regression functions from three sets of experiments were shown in **Figure 1B**. We found that the estimated half-life of PB1-F2 (PR8) calculated by regression analysis is 6.30 h, while that of PB1-F2 (HK156) is 2.14 h, suggesting that PB1-F2 (HK156) has a more rapid turnover rate. The longer protein half-life of PB1-F2 (PR8) may contribute to its higher protein production at zero time point since protein was allowed to accumulate for 16 h before cycloheximide was added. However, we cannot rule out the possibility that transcript of FLAG-PB1-F2 (PR8) can be translated more efficiently than that of FLAG-PB1-F2 (HK156).

### Mapping Residues Affecting Protein Half-life

**FIGURE 2:**
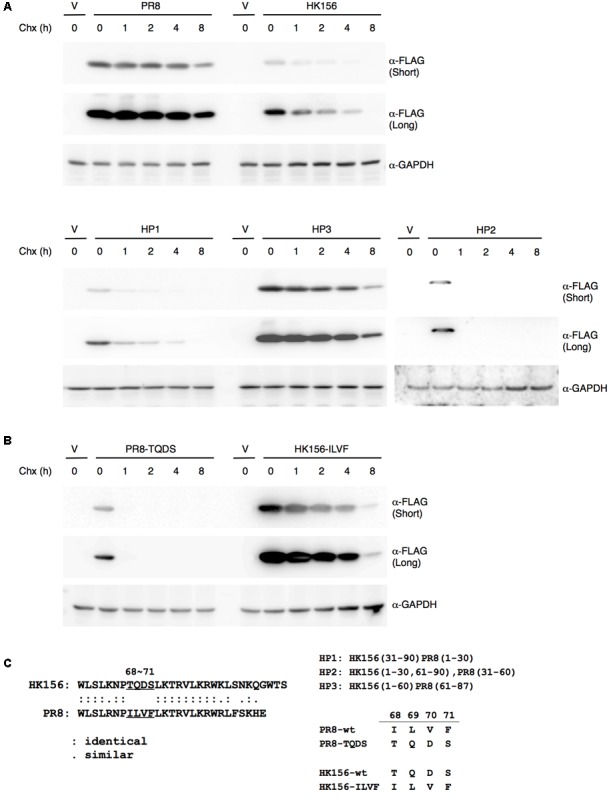
**Mapping motif affecting PB1-F2 half-life. (A)** Individual expression plasmid for FLAG-tagged PB1-F2 of HK156 and PR8, as well as for chimeric PB1-F2 (HP1), PB1-F2 (HP2) and PB1-F2 (HP3) was transfected into HEK293T cells for 16 h before cycloheximide (50 μg/ml) was added. Cells were harvested at 0, 1, 2, 4, and 8 h post-cycloheximide treatment. Western analysis was carried out using anti-FLAG antibody. β-actin served as a loading control. **(B)** Plasmids expressing FLAG-HK156-ILVF and FLAG-PR8-TQDS were transfected into HEK293T cells. Cycloheximide chasing assay and Western analysis were performed as in **(A)**. The shorter and longer exposures were both shown. V, vector control. **(C)** An amino acid sequence alignment between the last third of PB1-F2 (HK156) and PB1-F2 (PR8) was shown in left panel. Regions of PB1-F2 (PR8) and PB1-F2 (HK156) contained in chimeric PB1-F2, as well as sequence of amino acid 68–71 presenting in wild-type and mutant PB1-F2 were shown in right panel.

Since the protein half-life of PB1-F2 (HK156) is three-times shorter as that of PB1-F2 (PR8), we asked if there are particular amino acid residues responsible for this result. Alignment between two proteins reveals 57.8% identity and no apparent domains may contribute to the difference. We therefore constructed plasmids expressing chimeric proteins and measured their half-lives. Used plasmid expressing PB1-F2 (HK156) as the base, coding sequence for the first, second or last third of the protein was replaced with that of PB1-F2 (PR8), resulting pFLAG-HP1, pFLAG-HP2 and pFLAG-HP3, respectively. All three plasmids expressing chimeric FLAG-PB1-F2 were transfected into HEK293 cells. Cycloheximide chasing assay was performed as shown in **Figure 1**. We found the expression patterns of PB1-F2 (HP1) and PB1-F2 (HP2) are similar to that of PB1-F2 (HK156), while that of PB1-F2 (HP3) resembled the expression pattern of PB1-F2 (PR8) (**Figure 2A**). The results indicated the last 1/3 of protein containing residues determining protein half-life of PB1-F2. Within this region, sequence of amino acid 68–71 is completely different between PB1-F2 (HK156) and PB1-F2 (PR8). We therefore constructed two plasmids to produce PB1-F2 with these four amino acids swapped. Resulting plasmids pFLAG-HK156-ILVF and pFLAG-PR8-TQDS were transfected into HEK293 cells. Cycloheximide chasing assay was performed as in **Figure 2A**. We found that PB1-F2 (HK156-ILVF) now had an expression pattern similar to that of PB-F2 (PR8), while PB1-F2 (PR8-TQDS)’s resembled that of PB1-F2 (HK156), suggesting that amino acids 68–71 are the key residues affecting protein half-life of PB1-F2 (**Figure 2B**). A sequence alignment between the last third of PB1-F2 (HK156) and PB1-F2 (PR8), as well as the sequence of amino acids 68–71 in different constructs are shown in **Figure 2C**. It worth to note that PB1-F2 (HP2) may have a faster degradation rate than PB1-F2 (HK156), suggesting the middle third or PB1-F2 may contain residues contributing to its protein stability.

### Sensitivity of PB1-F2 Variants to MG132

**FIGURE 3:**
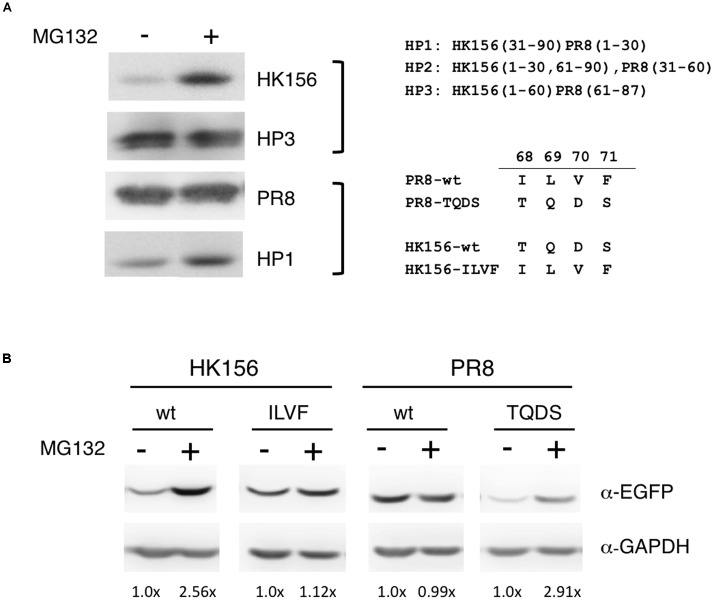
**Sensitivity to MG132 of PB1-F2 variants. (A)** Individual expression plasmid for FLAG-tagged PB1-F2 of HK156 and PR8, as well as for chimeric PB1-F2 (HP3) and PB1-F2 (HP1), was transfected into HEK293T cells for 6 h. MG132 (5 μM) was added for 16 h before cells were harvested for Western analysis using anti-FLAG antibody. **(B)** Individual expression plasmid for EGFP-tagged PB1-F2 of HK156 and PR8, as well as for mutants HK156-ILVF and PB1-F2-TQDS was transfected into HEK293T cells for 8 h. Cells with or without MG132 (5 μM) treatment were harvested 16 h later. Western analysis was carried out using anti-EGFP antibody. GAPDH served as loading controls. Chemiluminescence intensity for bands specific to EGFP-PB1-F2 and GAPDH were captured and measured. Signals with (+) or without MG132 (–) treatment were compared after normalized with GAPDH. Intensity of EGFP-PB1-F2 (MG132 (–)) after normalized with GAPDH was set as 1×. Constructs of chimeric PB1-F2 and sequence swapping mutants used in the experiments were shown.

Because one factor affecting protein turnover is how fast the protein is degraded, so we were interested in if variants and mutants of PB1-F2 are subjected to different degradation pathway. Others have shown that treatment of lactacystin, a proteasome inhibitor, increased the protein level of a PB1-F2 mutant but not its wild-type counter part (Schmolke et al., 2011), therefore we examined if various PB1-F2 proteins have equal sensitivities to proteasome-mediated degradation. Using the plasmids expressing FLAG-PB1-F2 of wild-type (PR8 and HK156) or chimeric (HP1 and HP3) proteins, we transfected HEK293 for 6 h before MG132, a proteasome inhibitor, was added. Cells were harvested and analyzed for protein levels of PB1-F2 in the presence or absence of MG132 by Western analysis. We found that MG132 treatment only increased levels of PB1-F2 of HK156 and HP1, but not those PR8 and HP3, indicating that the last 1/3 of PB1-F2 regulated proteasome-mediated PB1-F2 degradation (**Figure 3A**). Similarly we examined if amino acids 68–71 are responsible for this observation. Plasmids expressing EGFP-PB1-F2 were used to transfect HEK293 cells and experiments were carried out as in **Figure 3A**. We found that swapping of amino acids 68–71 changed its sensitivity to MG132. The increasing level by MG132 treatment for wild-type PB1-F2 (HK156) dropped from 2.56× to 1.12× when it came the ILVF mutant. On the other hand, the increasing level by MG132 treatment for wild-type PB1-F2 (PR8) increased from 0.99× to 2.91× when it came the TQDS mutant indicating that T68, Q69, D70 and S71 prompted PB1-F2 to proteasome degradation (**Figure 3B**). These results suggested that higher expression level of PR8-F2 (PR8) may be due to its resistance to proteasome degradation, and amino acids 68–71 (ILVF) is important for this resistance.

### Cellular Localization of Chimeric PB1-F2

**FIGURE 4:**
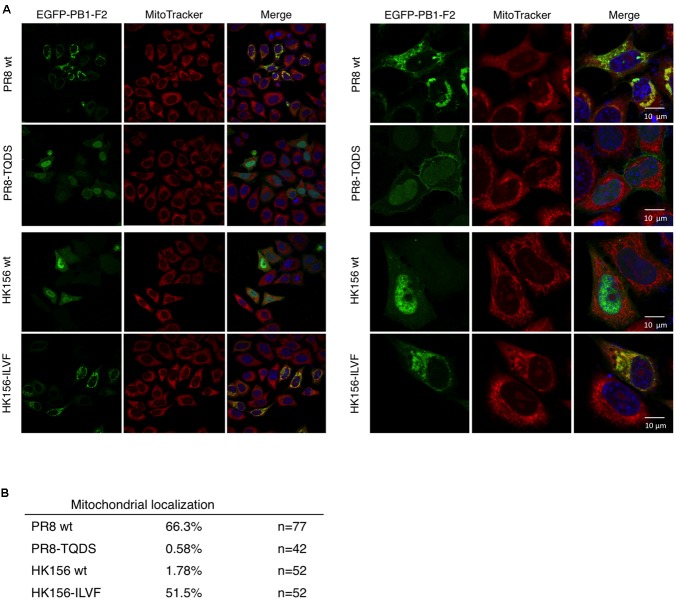
**Cellular localization by immunofluorescence confocal microscopy. (A)** Plasmids expressing EGFP-tagged PB1-F2 of HK156 and PR8, as well as HK156-ILVF and PR8-TQDS, two swapping mutants, were transfected into HeLa cells. Cells were stained with DAPI (blue) for nuclei and MitoTracker Orange (red) for mitochondria. EGFP signal indicates location of PB1-F2 protein (green). Yellow color indicates merge of red and green signals. Images were captured using Objective Plan-Apochromat 1.4 Oil DIC M27 with power of 63× (Left) or 100× (Right) with Zeiss LSM700. **(B)** Mitochondrial localization of denoted PB1-F2 was calculated as the percentage of signals of EGFP/MitoTracker. n, number of cells observed from 10 randomly selected fields.

When PB1-F2 (PR8) was first identified, it has a mitochondrial localization (Chen et al., 2001). Subsequent studies, on the other hand, demonstrated that PB1-F2 (HK156) localized mainly to the cytoplasm and nucleus (Chen et al., 2010). An optimal mitochondrial targeting sequence was identified to region 65–87 (Gibbs et al., 2003). Therefore we asked if swapping of amino acids 68–71 changes subcellular localization of PB1-F2. For this, plasmids expressing EGFP fusion protein with PB1-F2 of PR8, HK156, PR8-TQDS, or HK156-ILVF were constructed and transfected into HeLa cells. Cells were stained with MitoTracker for mitochondria and DAPI for nuclei. The fluorescence images showed that PB1-F2 (PR8) is localized to mitochondria, while PB1-F2 (HK156) is localized to cytosol and nuclei as predicted. However, amino acids 68–71 swapping between two proteins changed their cellular localization drastically. PB1-F2 (PR8) lost its mitochondrial localization and dispersed in cytosol and nucleus when amino acids 68–71 were changed from ILVF to TQDS (**Figure 4A**, PR8-TQDS); conversely when PB1-F2 (HK156) grained mitochondrial localization when amino acids 68–71 were switched from TQDS to ILVF (**Figure 4A**, HK156-ILVF). A quantitation of mitochondrial localization was shown in **Figure 4B**. These results indicated that amino acids 68–71 not only affect PB1-F2 protein stability, but also its cellular localization.

### IFN-β Modulation

**FIGURE 5:**
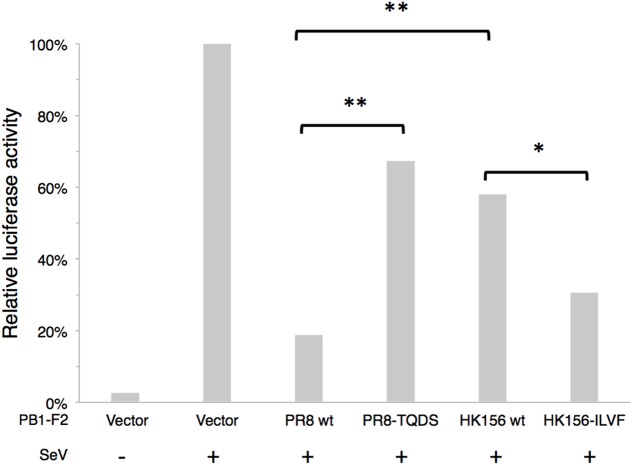
**IFN-β modulation by PB1-F2 variants.** Plasmids expressing PB1-F2 of PR8, HK156, PR8-TQDS, and HK156-ILVF were individually cotransfected with p125-luc, a reporter for IFN-β promoter activity, into HEK293T cells. 24 h post-transfection, cells were infected with Sendai virus (SeV) to induce IFN-β response. Infected cells were harvested 24 h post-infection for p125-luc reporter assay. Mean values of luciferase activity of two independent experiments with three readings each were shown. (^∗^*p* < 0.05; ^∗∗^*p* < 0.01, Student’s *t*-test).

It has been shown that PB1-F2 can modulate type I interferon (IFN) production through interfering RIG-I-mediated signaling at MAVS level (Varga et al., 2011). We asked if swapping of amino acids 68–71 can change this ability. Because NS1 of influenza virus is a potent inhibitor of RIG-I-mediated signaling pathway, we used SeV to induced IFN-β signaling cascade, which could be reflected by IFN-β reporter (p125-luc) activity. Plasmids expressing PB1-F2 was cotransfected with p125-luc reporter into HEK293 cells. 24 h post-transfection, cells were infected with SeV to induce IFN-β response. Infected cells were harvested 24 h post-infection for p125-luc reporter assay. SeV infection induced about 40-fold of p125-luc activity compared to non-infection control. PB1-F2 (PR8) caused an 81% drop of SeV-induced IFN-β promoter activity. On the other hand PB1-F2 (PR8-TQDS) only caused a 33% reduction. This is consistent with the decreased protein stability and reduced mitochondrial localization of this protein. Changing amino acids 68–71 from TQDS to ILVF further enhanced the ability of PB1-F2 (HK156) to inhibit p125-luc from 58 to 31% (**Figure 5**). This is consistent with the increased protein stability and mitochondrial localization of PB1-F2 (HK156-ILVF). These results indicated that amino acids 68–71 are key residues that affecting protein half-live and the functions of PB1-F2. Although PB1-F2 (HK156) did not show mitochondrial localization, it reduced SeV-mediated p125-luc activity to 58%. This may due to its inhibitory effect on NF-κB (Reis and McCauley, 2013), whose binding site also presents in the p125-luc promoter.

### Production of PB1-F2 during Infection

**FIGURE 6:**
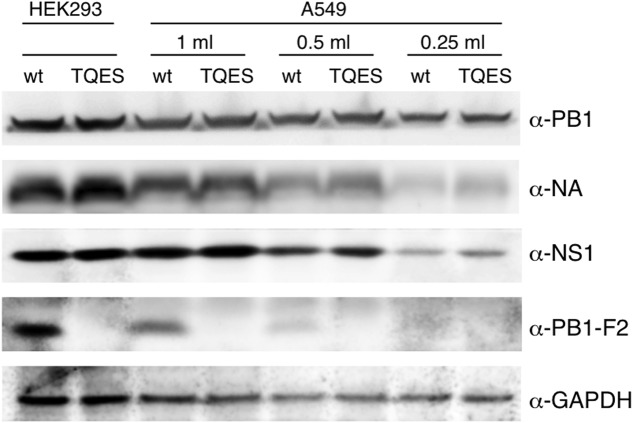
**Production of PB1-F2 during infection.** Recombinant A/Puerto Rico/8/1934 carrying PB1-F2 (68T, 69Q, 70E, 71S) was made using an eight-plasmids reverse genetics system. Plasmid pHW192 or pHW192-(PB1-F2 TQES) was transfected with seven other plasmids into HEK293 cells to generate PR8 wild-type (wt) virus or PR8 (PB1-F2-TQES) mutant virus, respectively. Transfected cells and supernatant were collected 48 h later. 2.5 × 10^5^ A549 cells were inoculated with 0.25 ml, 0.5 ml, and 1 ml of supernatants and harvested 24 h post-infection. Both transfected HEK293 and infected A549 cells were subjected to Western analysis for viral protein productions using antibodies indicated.

It is important to know if amino acid residues 68–71 effect PB1-F2 production during viral infection. We used PR8 virus as our model virus to address this question. Because PB1-F2 is encoded by the +1 ORF in the PB1 gene (Chen et al., 2001), we mutated the coding sequence from ILVF to TQES instead of TQDS to avoid changing the coding of PB1. Recombinant PR8 carrying PB1-F2 (T68, Q69, E70, S71) was made using an eight-plasmids reverse genetics system resulting the PB8 (PB1-F2-TQES) mutant virus in HEK293 cells. The supernatant was collected and used to inoculate A549 cells. Wild-type PR8 generated by reverse genetics served as a control. 24 h post-infection, cells were harvested for Western analysis. While PB1-F2 can be readily detected in A549 infected with PR8 virus, it was not detected in cells infected with PB8 (PB1-F2-TQES) mutant virus (**Figure 6**). This discrepancy is unlikely due to unequal inoculation since productions of other viral proteins, PB1, NA, and NS1 were similar in cells infected with different viruses. This result confirms our studies *in vitro* that amino acid residues 68–71 play an important role in PB1-F2 stability.

## Discussion

PB1-F2 was identified as a proapoptotic protein (Chen et al., 2001). Subsequent studies found it down-regulates interferon production (Varga et al., 2011, 2012) and up-regulates viral RNA polymerase activity (Mazur et al., 2008). Numerous studies demonstrated its function is host- and strain-specific (Varga and Palese, 2011). However, the mechanism underlying this property is not clear. Because an important factor affecting protein functions is its stability, we looked for the key sequence that determines the stability of PB1-F2. Compared PB1-F2 (HK156) to PB1-F2 (PR8), a variant with very different properties, and chimeric proteins between two, we identified amino acids 68–71 have important roles in protein stability, subcellular localization, and interferon deregulation. I68, L69, V70, and F71 of IAV PR8 strain permit higher stability of the protein (**Figures 1–3**), mitochondrial localization (**Figure 4**) and better interferon antagonization (**Figure 5**). On the other hand, T68, Q69, D70, and S71 of HK156 strain assert opposite effects on PB1-F2. We also demonstrated that PB1-F2 (PR8-TQES) has a much shorter protein half-life compared to its wild-type counterpart in PR8-infected cells (**Figure 6**). An important question is what underlies all these differences. Kosik et al. (2015) showed that ubiquitination of the K73/78/85 promoted PB1-F2 (PR8) degradation. Converting these lysine residues to arginine not only increased protein level, but also changed the properties of PB1-F2, such as subcellular localization, enhancement of vRdRp activity, and IFN-β antagonism (Kosik et al., 2015). Because conversion of amino acid residues 68–71 from TQDS to ILVF in PB1-F2 (HK156) rendered similar characteristics to PB1-F2 as observed in the K to R PB1-F2 mutants, it is possible these four residues have a role in regulating ubiquitination of the K73/78/85, therefore its protein half-life. However, when examined PB1-F2 (HK156) or PB1-F2 (PR8) with various lysine residues mutated to arginine, we did not find K to R mutation increased protein life of PB1-F2, suggesting that factors other than ubiquitination of lysine residues may affect PB1-F2 protein stability. Comparisons of protein half-life between PB1-F2 (HK156) and its 4KR mutant, as well as PB1-F2 (PR8) and its 7KR mutant, were shown in Supplementary Figure S1. However, we did find that PB1-F2 (PR8) could be colocalized with ubiquitin signals when cells were infected with SeV (Supplementary Figure S2). We do not know what made this discrepancy. We speculate that ubiquitination on PB1-F2 did not occur in our system without induction, which explains why we did not find that K to R mutation affected protein level of PB1-F2 in our hands. Structural studies of PB1-F2 have predicted that its C terminus has a high propensity for α-helix formation. It is interesting to note that region of amino acids 68–71 is part of an α-helix in PB1-F2 (PR8), while it is located in a linker region of two α-helices in PB1-F2 (HK156) in the predicted models. In addition, I68, L69, V70, F71, and L72 were shown to be important for the oligomerization state of PB1-F2 (PR8) (Bruns et al., 2007; Solbak et al., 2013). It is a possibility that swapping amino acid residues 68–71 between two PB1-F2 proteins led to changes of protein structure or oligomerization state of PB1-F2, and therefore affects its stability and functions.

Previous amino acid sequence alignments of PB1-F2 proteins revealed the uniqueness of I68, L69, V70, and F/S71 among influenza A viral strains expressing full-length of PB1-F2. The sequence is found in PR8, A/Taiwan/3355/1997 (H1N1), and A/William Smith Neurotropic/33 (H1N1, aka WSN), all H1N1 subtypes. On the other hand, T68, Q69, D/G70 and S71 are found in H3N2, H2N2, H5N1, and H6N1 subtypes (Chen et al., 2001; Alymova et al., 2014). Both PB1-F2 27-mer and full-length protein with I68, L69, and V70 caused more cell death than those without in these studies (Le Goffic et al., 2011; Alymova et al., 2014). Other than induce higher mitochondrial outer membrane permeabilization in previous studies (Le Goffic et al., 2011; Alymova et al., 2014), I68, L69, and V70 were shown here to be important for PB1-F2 protein stability and its mitochondrial localization. Our data added to the explanation that why PB1-F2 with I68, L69, V70, and F/S71 signature caused more cell death carried out by others. It has been shown that N66S mutation in the PB1-F2 of HK156 contributes to increased virulence (Conenello et al., 2007). We therefore checked if N66S increases PB1-F2 protein level in HEK293T and found it does not (data not shown). Because HK156 is an IAV with avian origin, we examined if PB1-F2 (HK156) may have a longer protein half-life in DF-1, a chicken fibroblast cell line. Our preliminary data indicated that HK156 PB1-F2 is degraded at a lower rate than in HEK293T cells (data not shown), which may contribute to its functions in avian species.

A/California/7/2009 (H1N1)pdm09-like virus (herein (H1N1)pdm), which emerged in 2009, caused the pandemic and has established its circulation among humans since. (H1N1)pdm encodes a putative 11-amino acid form of PB1-F2 due to a stop codon at position 12 in its ORF. There are two additional stop codons at positions 58 and 88. (H1N1)pdm virus expressing a full-length PB1-F2 (90 amino acids) generated by reverse genetics has a minimal impact on virulence in mice (Hai et al., 2010). In our own study, PB1-F2-expressing (H1N1)pdm did not induce apoptosis in U937 cells as PB1-F2 (PR8). Upon further investigation, we found the protein level of this rescued PB1-F2 protein, which contains T68, Q69, E70, and Y71, was extremely low and could only be detected in the presence of MG132 (data not shown). We ration that the low impact of PB1-F2 in animal or cell models is partially due to the unstable nature of this protein. It is interested to note that in several studies using PB1-F2-restored (H1N1)pdm, none of them showed the protein expression level of PB1-F2 (Hai et al., 2010; Ozawa et al., 2011).

In summary, we identified amino acid residues 68∼71 play important role in protein stability and functions of PB1-F2. Present study gives a reason to why PB1-F2 with I68, L69, and V70 signature can cause more cell death is due to better protein stability. Because the protein stability of PB1-F2 plays an important role in regulating its biological functions, we suggest that it is a major factor contributing to strain-specific and cell type-specific property of PB1-F2.

## Author Contributions

C-JC conceived the project, and together with Y-YC, prepared the manuscript. Y-YC, S-RY, Y-TW, and Y-HL designed and preformed the experiments.

## Conflict of Interest Statement

The authors declare that the research was conducted in the absence of any commercial or financial relationships that could be construed as a potential conflict of interest.
