# Supplementary material for: Amino Acid Residues 68–71 Contribute to Influenza A Virus PB1-F2 Protein Stability and Functions
Source: Front Microbiol. 2017 Apr 21;8:692. doi: 10.3389/fmicb.2017.00692 (PMC5399091; doi:10.3389/fmicb.2017.00692)
Supplement: Supplementary file 1 [file Data_Sheet_1.docx]

Supplementary Material

Amino acid residues 68 - 71 contribute to influenza A virus PB1-F2 protein stability and functions

Yi-Ying Cheng*, Shih-Rang Yang, Ying-Ting Wang, Yu-Hsin Lin, and Chi-Ju Chen

Correspondence: Corresponding Author: [cjchen@ym.edu.tw](mailto:cjchen@ym.edu.tw)

Materials and Methods for Fig. S1 and Fig. S2

Construct expressing plasmids for FLAG-tagged PB1-F2 (PR8-7KR) and PB1-F2 (HK156-4KR). Overlapping PCR extension cloning was used to generate lysine (K) to arginine (R) mutants with flanking primers No. 621 and No. 622. PR8-7KR is a K20, 29, 44, 53, 73, 78, 85R mutant, while HK156-4KR is a K65, 78, 81, 85R mutant. Internal primer design and the templates used to step-wise generate these two constructs are listed in Tables S1 and S2. All constructs were sequenced for accuracy.

Ubiquitination of PB1-F2 (PR8) by immunofluorescence microscopy. mCherry-Ub and PB1-F2 (PR8) were overproduced in HeLa cells. Cells were then infected with SeV (5 HAV) for 16 h and harvested. After permeabilization with 0.4% Triton X-100 in PBS for 5 minutes, cells were blocked with 5% BSA and incubated with anti-FLAG antibody, followed by FITC-labeled anti-mouse secondary antibody. Confocal microscopy was performed with a Zeiss LSM700 microscope.

Table S1. Primers and used for constructing PB1-F2 K to R mutants and PB1-F2 sequence comparison between wild-types and mutants.

| pFLAG-PB1-F2 (PR8-7KR) | Forward | Reverse | Template |
| --- | --- | --- | --- |
| K29R | No. 850 | No. 851 | pFLAG-PR8-PB1-F2 |
| K44R | No. 852 | No. 853 | pFLAG-PR8-PB1-F2 |
| K53R | No. 854 | No. 855 | pFLAG-PR8-PB1-F2 |
| K20, 29R | No. 864 | No. 863 | pFLAG-PR8-PB1-F2 K29R |
| K44, 53R | No. 866 | No. 865 | pFLAG-PR8-PB1-F2 K20R, K29R |
| K73R | No. 856 | No. 857 | pFLAG-PR8-PB1-F2 K20R, K29R, K44R, K53R |
| K73,78R | No. 860 | No. 861 | pFLAG-PR8-PB1-F2 K20R, K29R, K44R, K53R, K73R |
| K85R | No. 860 | No. 862 | pFLAG-PR8-PB1-F2 K20R, K29R, K44R, K53R, K73R, K78R |
| PR8-wt MGQEQDTPWI LSTGHISTQK REDGQQTPKL EHRNSTRLMG HCQKTMNQVV MPKQIVYWRR WLSLRNPILV FLKTRVLKRW RLFSKHE*  PB8-7KR .......... .........R ........R. .......... ...R...... ..R....... .......... ..R....R.. ....R... | | | |
| pFLAG-PB1-F2 (HK156-4KR) | Forward | Reverse | Template |
| K65R | No. 581 | No. 582 | pFLAG-HK156-PB1-F2 |
| K81R | No. 583 | No. 584 | pFLAG-HK156-PB1-F2 |
| K78R | No. 669 | No. 670 | pFLAG-HK156-PB1-F2 K65R |
| K81R, K85R | No. 621 | No. 678 | pFLAG-HK156-PB1-F2 K65R, 73R, K78 |
| \| HK156-wt MEQEQDTPWT QSTEHINIQK KGGGQQTQRP EHPNSTLLMD HYLKITSRAG MHKQIVYWKQ WLSLKNPTQD SLRTHVLKRW KLSSKQEWTN * HK156-4KR .......... .......... .......... .......... .......... .......... ....R..... .......R.. R...R..... . \| \| --- \| | | | |

Table S2. Primer sequences used for constructing PB1-F2 K to R mutants

| pFLAG-PB1-F2 (PR8-7KR) | |
| --- | --- |
| Primer | Sequence |
| No. 864 | ACATCAGTACTCAG**AGA**AGGGAAGATGGACAACAAACACCG**AGA**CTGGAGCACCGCAA |
| No. 863 | TTGCGGTGCTCCAG**TCT**CGGTGTTTGTTGTCCATCTTCCCT**TCT**CTGAGTACTGATGT |
| No. 866 | TGGGCCACTGCCAG**AGG**ACAATGAACCAAGTGGTTATGCCC**AGA**CAGATTGTGTATTG |
| No. 865 | CAATACACAATCTG**TCT**GGGCATAACCACTTGGTTCATTGT**CCT**CTGGCAGTGGCCCA |
| No. 850 | GGACAACAAACACCG**AGA**CTGGAGCACCGCAAC |
| No. 851 | GTTGCGGTGCTCCAG**TCT**CGGTGTTTGTTGTCC |
| No. 852 | GATTGATGGGCCACTGCCAG**AGG**ACAATGAACCAAGTGGTTAT |
| No. 853 | ATAACCACTTGGTTCATTGT**CCT**CTGGCAGTGGCCCATCAATC |
| No. 854 | TGAACCAAGTGGTTATGCCC**AGA**CAGATTGTGTATTGGAGGCG |
| No. 855 | CGCCTCCAATACACAATCTG**TCT**GGGCATAACCACTTGGTTCA |
| No. 856 | CCATCCTGGTATTTTTG**AGA**ACTCGTGTATTGAAACG |
| No. 857 | CGTTTCAATACACGAGT**TCT**CAAAAATACCAGGATGG |
| No. 860 | CATCCTGGTATTTTTG**AGA**ACTCGTGTATTG**AGA**C |
| No. 861 | G**TCT**CAATACACGAGT**TCT**CAAAAATACCAGGATG |
| No. 862 | CGATGAATTCCTACTCGTG**TCT**GCTGAACAACCTCCATCG |
|  | |
| pFLAG-PB1-F2 (HK156-4KR) | |
| No. 581 | GGAAGCAATGGCTTTCCTTG**AGG**AATCCCACCCAGGACTC |
| No. 582 | TCCTGGGTGGGATT**CCT**CAAGGAAAGCCATTGCTTCCAAT |
| No. 583 | CATGTCTTGAAACGAT**GGA**GGTTGTCCAGCAAACGAG |
| No. 584 | CTCGTTTGCTGGACAA**CCT**CCATCGTTTCAAGACATGAGT |
| No. 669 | GAACTCATGTCTTG**AGA**CGATGGAAGTTGTC |
| No. 670 | GACAACTTCCATCG**TCT**CAAGACATGAGTTC |
| No. 678 | CGATGAATTCTTAGTTTGTCCACTCTTG**TCT**GCTGGACAA**CCT**CCATC |

Bold letters indicate the coding for arginine (R); red letters indicate mutated nucleotides.


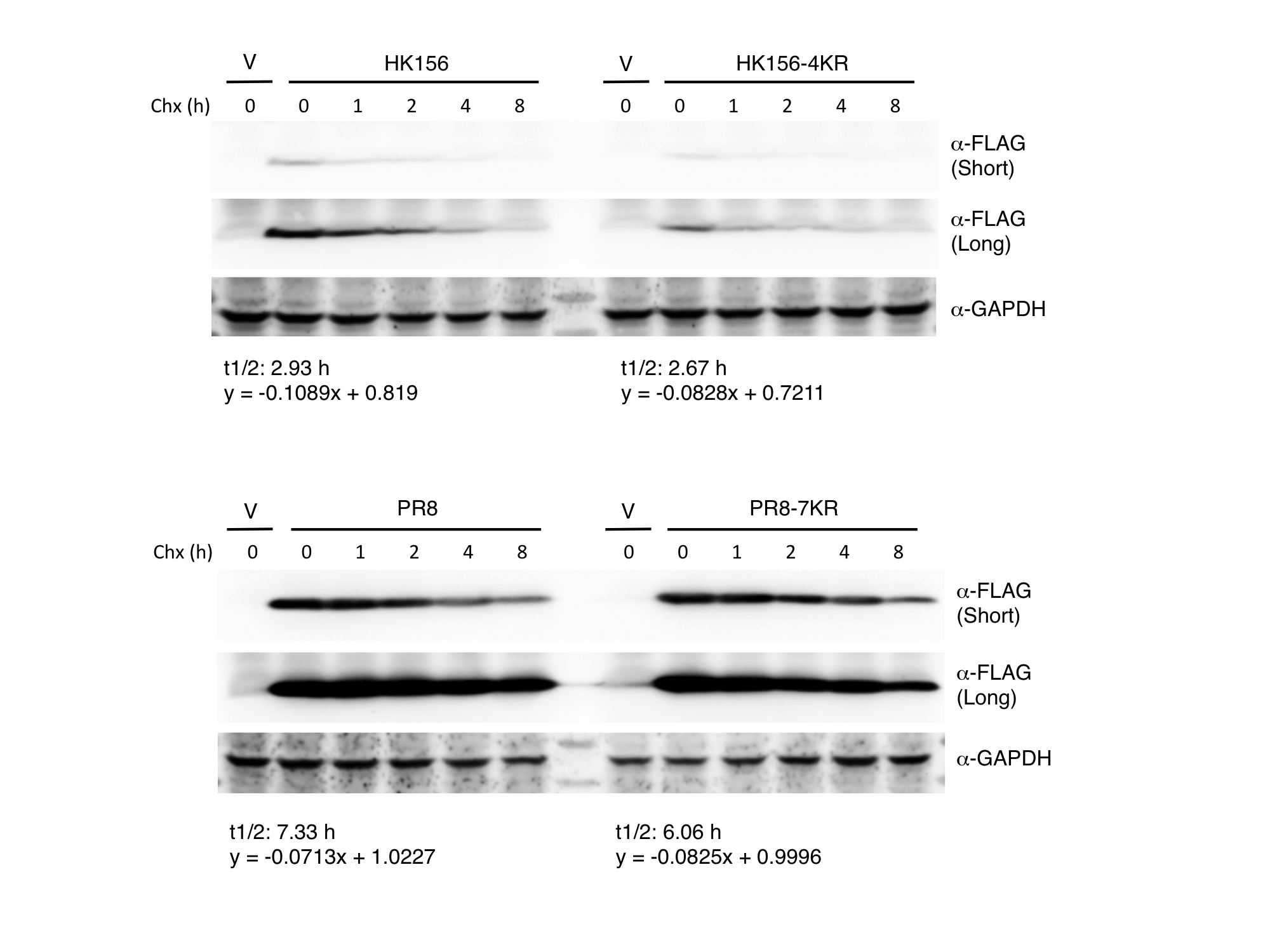


Figure S1. Effects of K to R mutation on PB1-F2 half-life. Plasmids expressing FLA-HK156, FLAG-HK156-4R, FLAG-PR8, and FLAG-PR8-7R were transfected into HEK293T cells. or 16 h before cycloheximide (50 µg/ml) was added. Cells were harvested at 0, 1, 2, 4, and 8 h post-cycloheximide treatment. Western analysis was carried out using anti-FLAG antibody. GAPDH served as a loading control. PR8-7KR is a K20, 29, 44, 53, 73, 78, 85R mutant, while HK156-4KR is a K65, 78, 81, 85R mutant. Intensity for bands specific to PB1-F2 at each time point was measured by densitometer and plotted. Protein half-life (t1/2) and regression formulation calculated for each PB1-F2 were indicated.


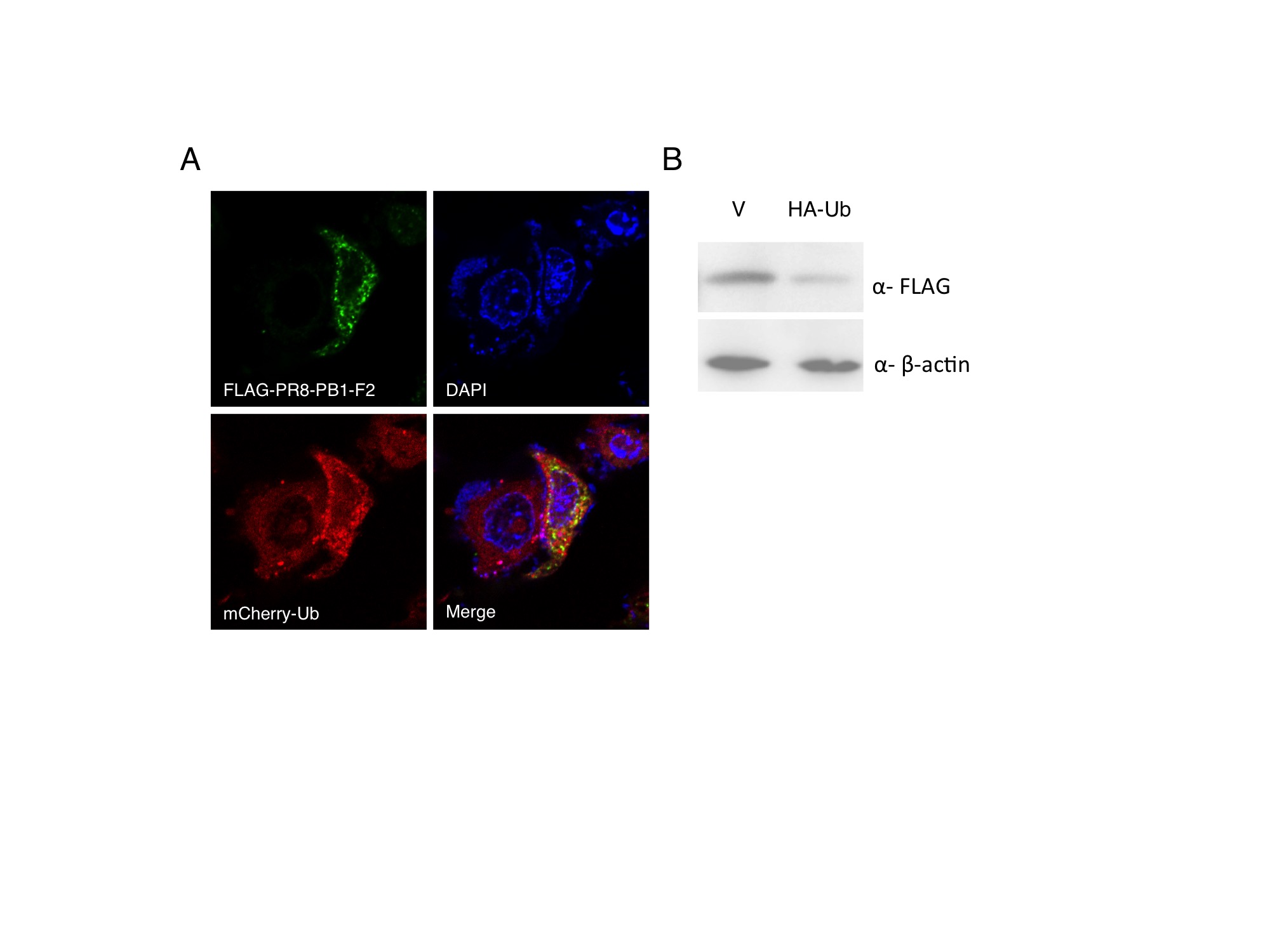


Figure S2. PB1-F2 (PR8) colocalized with ubiquitin signals. mCherry-Ub and FLAG-PB1-F2 (PR8) were over produced I HeLa cells. Cells were infected with SeV (5 HAU) in these cells for 16 h before harvesting for (A) immune fluorescence confocal microscopy and (B) Western analysis using antibodies indicated. V: vector control; HA-Ub: Ha-Ub was overexpressed.
